# Simulated environmental weathering of expanded polystyrene foam and polypropylene under UV and wave agitation

**DOI:** 10.1038/s41598-025-22367-7

**Published:** 2025-11-04

**Authors:** Sucheela Polruang, Varinporn Asokbunyarat, Phichayut Bouthong, Fauzul Rizqa, Anchisa Somprasong

**Affiliations:** Department of Environmental Engineering, Faculty of Engineering, Kasetsart University, Bangkok, 10900 Thailand

**Keywords:** Plastic weathering, Microplastic formation, Photo-oxidative degradation, UV exposure effects, Water waves, Expanded polystyrene (EPS), Polypropylene (PP), Environmental sciences, Ocean sciences

## Abstract

Improper plastic waste management contributes to environmental accumulation and poses significant risks to marine ecosystems and human health. Once in aquatic environments, plastics undergo weathering, leading to physical and chemical changes and fragmentation into microplastics (MPs). This study examines the weathering of expanded polystyrene (EPS) foam containers and polypropylene (PP) bags under ten simulated conditions including UV exposure in dry and wet environments (seawater and deionized water), with and without artificial waves. Surface changes were assessed using stereo microscopy, scanning electron microscopy (SEM), and Fourier transform infrared spectroscopy (FTIR). EPS foam exhibited discoloration, surface cracking, and fragmentation, particularly under UV exposure. These effects were pronounced in both dry and wet environments and confirmed by SEM imaging. PP showed gradual surface changes and fragmentation only under UV exposure with wave agitation, mainly in dry and seawater conditions, without clear discoloration. Carbonyl index (CI) analysis indicated more advanced chemical degradation in EPS foam compared to PP. FTIR spectra revealed new peaks corresponding to C = O and O–H bonds, consistent with photo-oxidative degradation. These results highlight material-specific weathering behaviors and underscore the roles of UV radiation and hydrodynamics in accelerating secondary MP formation, emphasizing the need for comprehensive plastic waste management strategies.

**Supplementary Information:**

The online version contains supplementary material available at 10.1038/s41598-025-22367-7.

## Introduction

Solid waste mismanagement remains a significant global issue, contributing to pollution, ecosystem degradation, and human health risks. As of mid-2024, the global population has reached nearly 8.2 billion and is projected to grow to 10.3 billion by the mid-2080s^1^. This growth, combined with urbanization, is expected to increase solid waste production to 9.3 billion tons by 2050 ^2,3^. Over the past five decades, approximately 12,000 million tons of plastic waste have entered the environment, primarily from countries with inadequate waste management^4^. While municipal solid waste (MSW) generation may peak around 2029 before declining by 2035, ineffective policies could lead to severe environmental and socio-economic consequences^5,6^, threatening progress toward the UN Sustainable Development Goals (SDGs), particularly Goals 11, 12, and 14.

A major consequence of poor waste management is plastic accumulation in aquatic environments, with over 80% of marine plastic litter originating from land^7–9^. Once in the environment, plastic products undergo physical and chemical transformations, forming microplastics (MPs)^10–12^. MPs (< 5 mm) are classified as primary (intentionally manufactured) and secondary (fragmented from larger debris). Secondary MPs originate from sources such as mismanaged waste, untreated wastewater, road dust, motor vehicles, and stormwater runoff^8^. MPs have been detected in rivers, beaches, estuaries, and oceans^13–17^. These particles accumulate in aquatic food chains, posing risks to marine organisms and human health^18–20^. Additionally, MPs can adsorb and release toxic chemicals such as PAHs, phthalates, and heavy metals^21,22^, amplifying environmental hazards.

The degradation of plastic debris into secondary MPs is driven by abiotic factors such as UV radiation, temperature fluctuations, humidity, and mechanical abrasion^23,24^. UV radiation plays a key role in photo-oxidative degradation, while mechanical forces from water waves and salinity accelerate degradation^25^.

Among marine plastic debris, polypropylene (PP) and expanded polystyrene (EPS) foam are frequently detected due to their widespread use in packaging^26^. EPS foam is commonly used for disposable food containers and cups, while PP is widely used in single-use bags and packaging films. Both materials are prone to mismanagement, littering, and long-distance transport due to their lightweight properties, making them environmentally problematic.

Previous studies have examined plastic degradation under UV exposure^27–29^; however, research on the combined roles of UV radiation, mechanical water waves, and different aqueous conditions (seawater vs. deionized water) remains limited. Most studies focus on isolated factors, use different plastic products, or apply varying experimental setups, leaving gaps in understanding how these environmental conditions collectively influence degradation outcomes. Few studies have comprehensively explored these effects using PP and EPS foam from the same production batch under controlled conditions—an approach that enables meaningful comparisons within a single experimental framework.

This study systematically investigates PP and EPS foam degradation over six months under ten distinct controlled conditions, examining photodegradation (with and without UV), hydrodynamic forces (with and without mechanical waves), and aquatic environments (seawater vs. deionized water). By integrating these factors, it enhances understanding of plastic weathering mechanisms, supporting waste management strategies, microplastic pollution mitigation, and policy recommendations to reduce plastic waste impacts on marine ecosystems.

## Materials and methods

### Samples and experimental setup

#### Plastic samples

Two commonly used food packaging materials—expanded polystyrene (EPS) foam containers and polypropylene (PP) plastic bags—were obtained from a local supermarket (Brand: ARO). Both materials were cut into 1 × 1 cm pieces for the experiments. Before testing, the EPS foam containers were 4 mm thick with a rough, porous white surface, while the PP plastic bags consisted of a 0.06 mm thick smooth, colorless, transparent film (Fig. 3).

#### Experimental setup

**Fig. 1:**
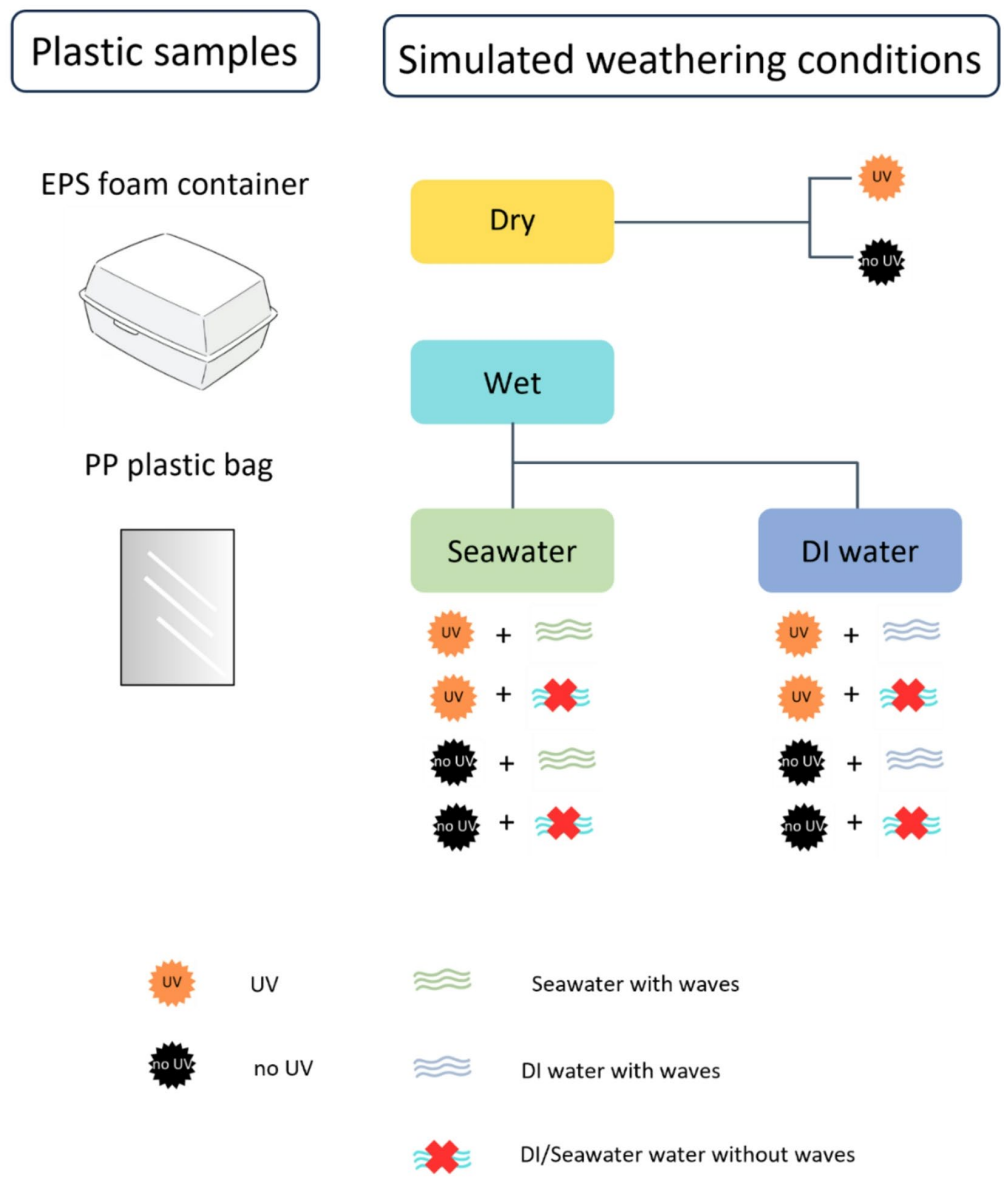
Experimental setup for both polymer types.

As illustrated in Fig. 1, the experiment simulated two main conditions: with and without UV exposure. Each condition was further divided into dry and wet environments. For each condition, 12 pieces of each plastic type (EPS foam and PP) were tested. In dry conditions, samples were placed evenly in 9 cm glass Petri dishes.

For wet conditions, two water treatments were tested: with and without wave agitation. Plastic samples were immersed in (i) seawater and (ii) deionized (DI) water. Seawater was collected from Laem Mae Pim Beach (Rayong Province, Thailand), filtered sequentially through 5,000, 300, and 20 μm meshes to remove macro- and microparticles, stored at 4 °C and brought to room temperature prior to use.

For the static wet condition (without water waves), samples were submerged in 0.035 m radius glass beakers filled with 150 mL of the corresponding solution and kept in a steady reactor. In the dynamic wet condition (with water waves), samples were submerged in identical beakers with the same solution volume and subjected to rotary shaker agitation.

The shaker’s rotation speed was set at 150 rpm, determined from the average wave speed in the Gulf of Thailand, which varies between 2 and 9 m/min during the dry season (November–April) and 3–11 m/min during the wet season (May–October)^30^. Under certain weather conditions, tides and temperature differences can cause wave speeds to reach 80–100 m/min. To simulate a more intense hydrodynamic environment, the experimental water wave speed was set to three times the maximum all-year average, approximately 33 m/min.

The corresponding rotation speed (f) of the shaker was calculated using the equation:

1 $$f=v/{\text{2p}}r$$

where *f* is the frequency of water waves or the shaker’s rotation speed (min^− 1^ or rpm), *v* is the water wave speed (33 m/min), *r* and is the radius of the beakers (0.035 m).

**Fig. 2:**
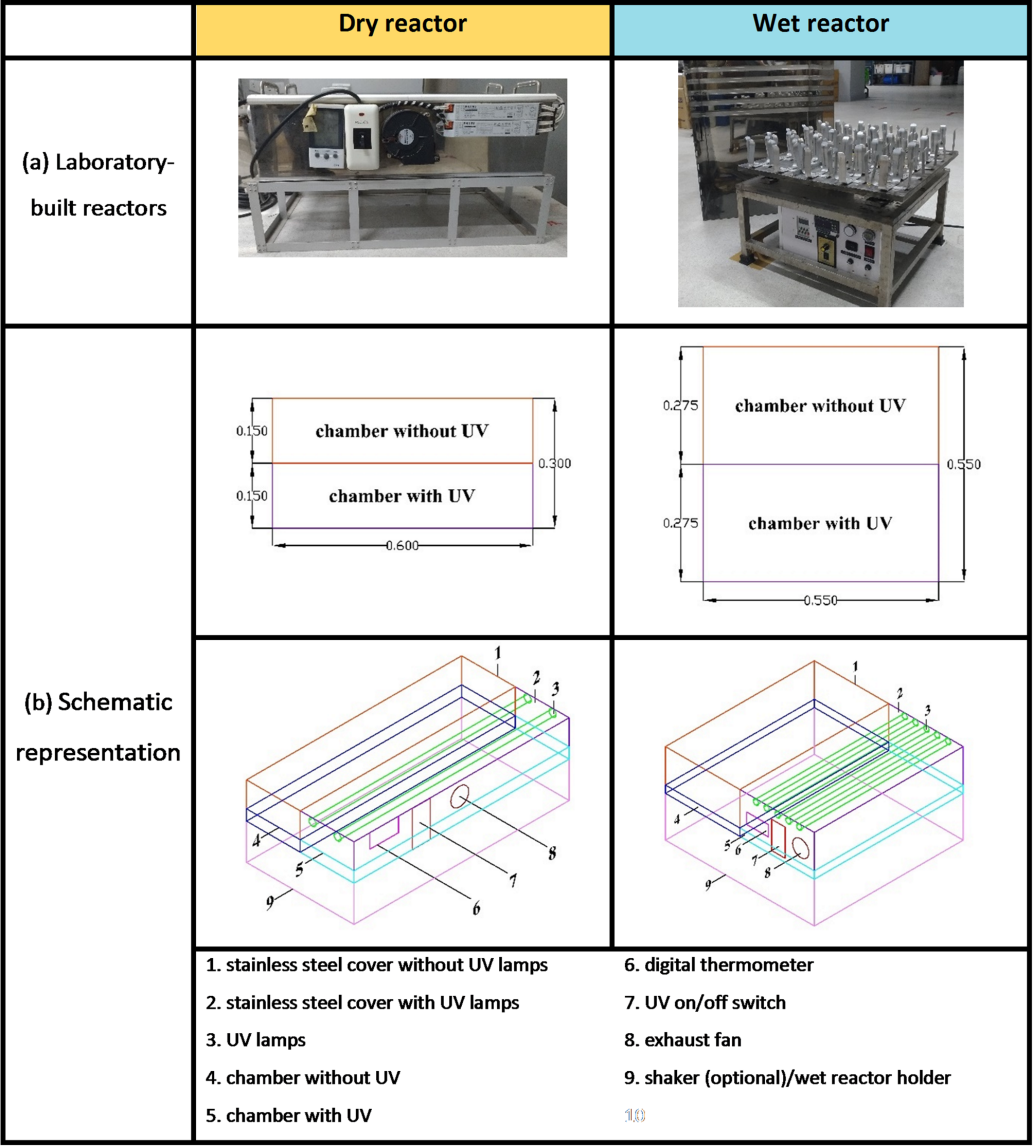
(**a**) Photographs of the laboratory-built reactors used in the experiments. (**b**) Schematic representation of the reactor design, showing key components.

As illustrated in Fig. 2, the setup included three custom-built laboratory reactors: one dry reactor (Fig. 2 (left)) and two wet reactors (Fig. 2 (right)), differentiated by the presence or absence of wave motion. The dry reactor measured 0.6 × 0.3 × 0.1 m, while both wet reactors measured 0.55 × 0.55 × 0.3 m. Each reactor had two chambers: one exposed to UV light and one shielded from UV by a stainless-steel cover to ensure darkness.

UVA exposure was calibrated using a Gigahertz-Optik P‑9710‑1 photometer (detection range 0–20 W/m², accuracy ± 4%). Natural sunlight measurements were taken five times daily (8:00, 10:00, 12:00, 14:00, 16:00) over five randomly selected days in July 2023 under varying weather conditions (cloudy, lightly cloudy, lightly sunny, very sunny). The average UVA intensity was 8 W/m², consistent with The World Bank Group’s reported range (8–16 W/m²)^31^. This average value was used to set the reactor UVA intensity at ~ 12 W/m²—about 1.5× peak daytime UVA. UVA irradiation was supplied by five Philips TL‑D 36 W BL fluorescent lamps (peak 365 nm), delivering ~ 12 W/m² at the sample surface. Samples were exposed continuously (24 h/day) for 180 days (6 months). Fans were used to maintain the reactor temperature between 30 and 35 °C throughout the experiment, reflecting typical daytime conditions in tropical coastal areas such as the Gulf of Thailand^30^.

### Analysis and characterizations

Samples were collected at months 0, 2, 4, and 6 (M0, M2, M4, and M6). At each time point, nine pieces were randomly selected from every condition to evaluate physical appearance, surface morphology, and chemical structure. Three of these pieces were allocated for each type of analysis. Wet samples were dried at room temperature and wrapped in aluminum foil to prevent further changes before testing.

The physical appearance of the plastic samples was examined using a stereomicroscope (Olympus SZ61) to document changes in surface texture and color.

Surface morphology was examined by scanning electron microscopy (SEM) with an FEI Quanta 450 microscope at 1000× magnification. Due to the heterogeneous textures of weathered surfaces, at least three regions were imaged for each piece to capture both smooth and rough areas^32^.

Prior to SEM analysis, all samples were rinsed thoroughly with deionized water to remove debris and salt residues. Seawater-exposed samples with visible salt crystallization were additionally cleaned in an ultrasonic bath (40 kHz, 3 min) to remove adhered salts following^33^. The cleaned samples were then air-dried at room temperature (~ 25–27 °C) in a dust-free chamber for at least 24 h. This pre-treatment protocol aligns with established SEM sample preparation techniques for polymeric materials, where air-drying is used to preserve surface features while avoiding structural distortion caused by specialized equipment such as critical-point dryers^34^.

Fourier Transform Infrared (FTIR) spectroscopy was used to analyze chemical structure changes with a Bruker Alpha-E FTIR equipped with an ATR module. Spectra were collected over 4000–1000 cm⁻¹. The Carbonyl Index (CI) was calculated as the ratio of the absorbance at ~ 1710 cm⁻¹ (carbonyl stretching region) to that at ~ 1465 cm⁻¹ (CH₂ bending vibration), following established methods in plastic weathering research^35–38^. CI values were calculated from at least three replicated per condition at each time point, reported as mean ± standard deviation. The absorbance in the carbonyl region may include overlapping contributions from various C = O-containing functional groups such as ketones, aldehydes, esters, carboxylic acids, and lactones. However, no peak deconvolution was performed. Therefore, the CI serves as a semi-quantitative indicator of total carbonyl group formation rather than identification of individual species. This approach allows for consistent comparisons of overall surface oxidation among different weathering conditions.

## Results and discussion

### Changes in appearance

**Fig. 3:**
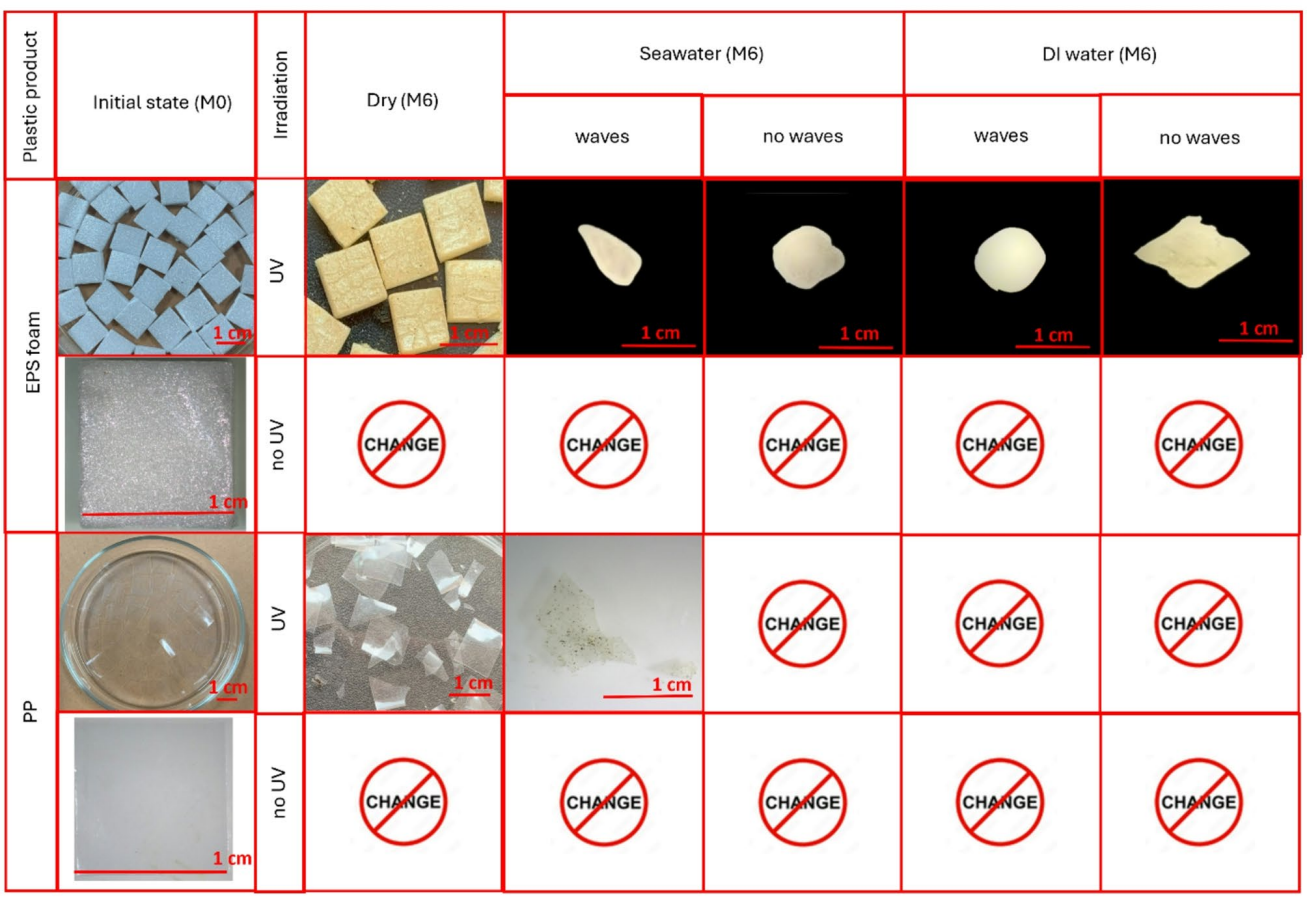
Changes in the appearance of EPS foam and PP in all experimental conditions comparing the initial state (M0) to the final state (M6).

Figure 3 illustrates the temporal changes in the appearance of EPS foam containers and PP plastic bags, comparing their initial state (M0) to the final state at the end of the experiment (M6).

**For EPS foam containers**, three primary changes were observed under UV exposure across all dry and wet conditions (seawater and DI water, with and without water waves): color change, surface cracking, and fragmentation. However, not all samples exhibited all of these changes.

Color change occurred in all UV-exposed EPS foam samples, transitioning from white (M0) to yellow (M6), as shown in Fig. 3. Observations at intermediate stages (M2 and M4) revealed a gradual darkening of the foam, progressing from pale yellow to deep yellow. This color change is typically associated with chemical alterations during degradation. Most polymers undergo yellowing over time due to photo-oxidation, where chromophores in the polymer chains absorb UV wavelengths above 290 nm, initiating degradation^40,41^. The extent of yellowing depends on factors such as the type and concentration of additives, as well as the duration of UV exposure^42^.

Surface cracking was observed only under specific conditions. In dry environments with UV exposure, the EPS foam surface developed numerous cell-like structures. Initially, hairline cracks formed (M2), which later evolved into more pronounced fissures (M4 and M6), indicating gradual surface deterioration without fragmentation. Fragmentation occurred solely in wet conditions with UV exposure, whether or not water waves were present. In these conditions, the samples exhibited thinning and shrinkage, with some deforming from square to trapezoidal shapes before fracturing into smaller pieces. In contrast, EPS foam samples that were not exposed to UV radiation showed no noticeable changes in appearance, regardless of whether they were in dry or wet environments.

**PP plastic bags** exhibited no color changes over six months in any environment, regardless of whether conditions were dry or wet, with or without UV exposure (see Fig. 3). However, progressive physical changes were noted. Under dry conditions with UV exposure, visible cracks began to form by M4, leading to fragmentation into smaller pieces by M6. In seawater with UV exposure and water waves, PP samples became brittle by M4, and some pieces disintegrated by M6. This fracturing is indicative of photo-oxidative degradation due to UV exposure^43^. No significant changes were observed in PP samples kept in dry environments without UV exposure, similar to the behavior of EPS foam under the same conditions. In all other wet conditions, except for seawater with UV exposure and water waves, PP samples remained largely unchanged over the six-month period.

### Changes in surface morphology

**Fig. 4:**
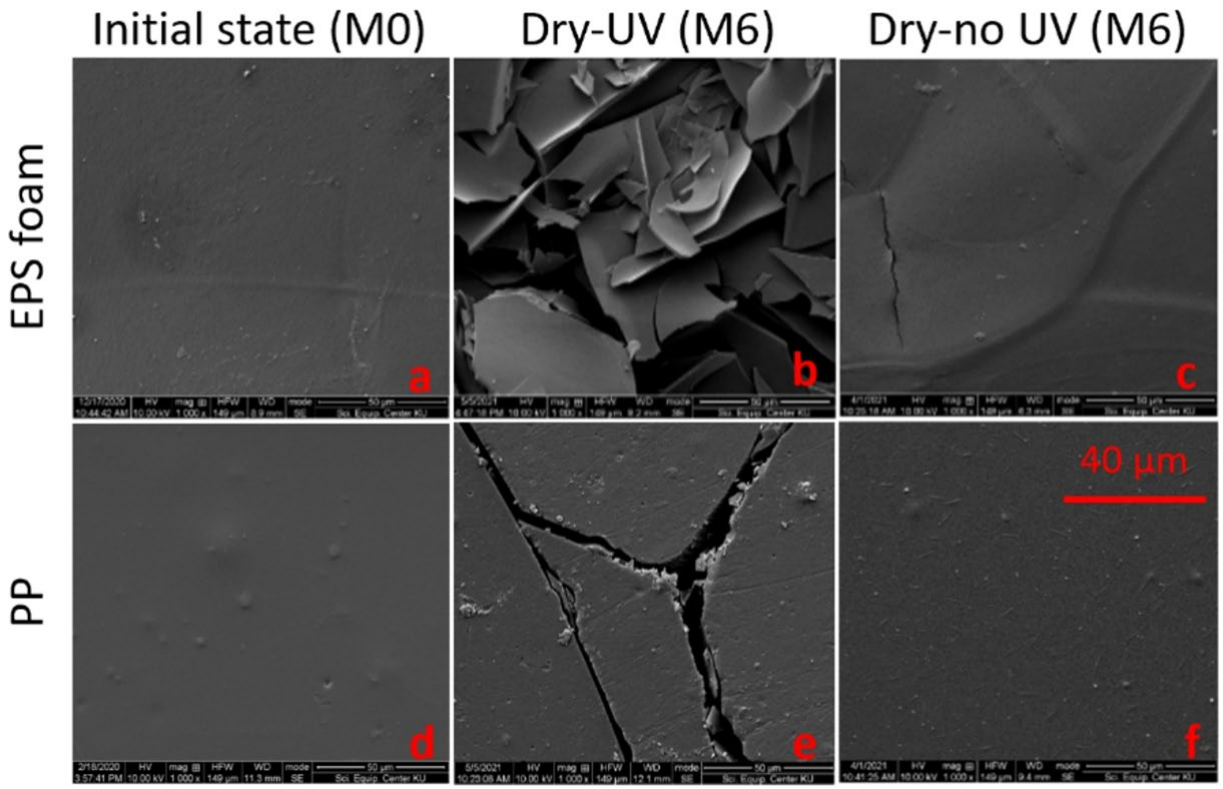
SEM micrographs of EPS foam and PP showing surface degradation after six months of exposure in simulated dry environments.

SEM images are useful for observing and evaluating changes in the surface morphology of plastic materials. As shown in Fig. 4a and d, the surface morphology of the unweathered plastic products at the initial state (M0) appeared relatively homogeneous, uniform, and smooth. By the final state (M6), an increase in surface roughness, flakiness, pits, and cracks was observed, indicating significant changes in the weathered plastic surfaces. Under dry environmental conditions and UV exposure, the surface morphology of EPS foam began to exhibit brittleness and flakiness by M4. These changes became progressively more pronounced and reached their most severe state by M6 (Fig. 4b). By contrast, Fig. 4c shows EPS foam without UV after six months, where only a fine, isolated crack is visible. Concurrently, the surface of the PP plastic bag displayed noticeable cracks after M4 of UV irradiation in a dry environment, which became more pronounced as the weathering process continued for up to six months. (Fig. 4e). By comparison, Fig. 4f shows PP without UV after six months, where no cracks or flakes are present. Flakes and cracks are commonly observed patterns of photo-oxidative degradation^32,44–46^. In particular, in a dry environment and UV exposure, after initial flakes and cracks were obtained on the surface of the EPS foam, they grew faster than those of the PP plastic bags. This may be because EPS foam has a higher amount of branch or side groups in polymer chains, which are responsible for an increase in gas permeability and lead to a higher rate of oxidative degradation^40,47^.

**Fig. 5:**
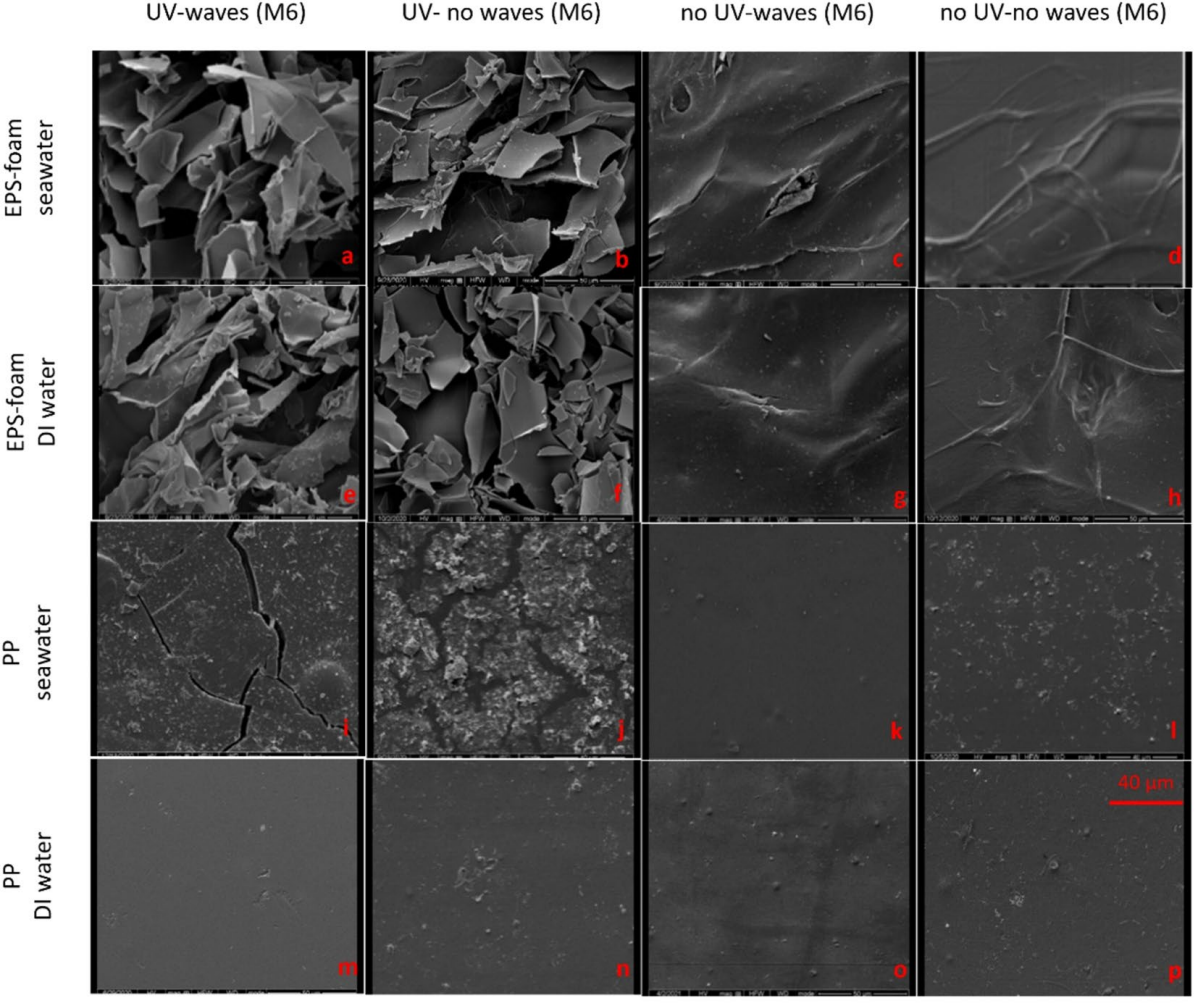
SEM micrographs of EPS foam (**a**–**h**) and PP (**i**–**p**) showing surface degradation after six months of exposure in simulated wet environments.

When subjected to a wet environment (seawater and DI water) under UV exposure for 6 months, the surface of EPS foam exhibited heterogeneity. Flaking, pitting, linear track marks, and creases were observed on the weathered EPS foam surface. The creases and crevices indicate shrinkage and compactness during the weathering process with UV (Fig. 5a and b; e and f). Under almost all dark conditions, SEM images of EPS foam after six months revealed minor morphological changes on the surface, primarily in the form of wrinkles (Fig. 5d; g–h). This excludes EPS foam in seawater without UV but with water waves, where initial cracks and wrinkles became evident in the SEM image (Fig. 5c).

This observation suggests that mechanical forces alone—such as continuous wave motion and water turbulence—can initiate physical degradation of EPS foam, even in the absence of UV radiation. The early-stage surface cracking and wrinkling observed in Fig. 5c may result from repeated mechanical abrasion and flexing of the polymer structure in seawater. While UV light is commonly regarded as the dominant initiator of plastic weathering, results highlight the important role of mechanical forces in initiating degradation, particularly for low-density, porous materials like EPS foam. Similar findings have been reported in recent studies on mechanical fragmentation and abrasion of plastics in aquatic environments, where wave-induced shear stress and particle collision contribute to surface weakening and fragmentation^48,49^.

In contrast, no discernible changes in surface morphology were observed on the PP plastic bags after six months (Fig. 5k–p), except under seawater conditions with UV exposure and water waves, where small cracks were observed (Fig. 5i). It should be noted that the stains observed on the SEM images of PP in seawater may correspond to salt flakes from seawater that were not completely removed before analysis (Fig. 5j).

### Changes in FTIR spectra

**Fig. 6:**
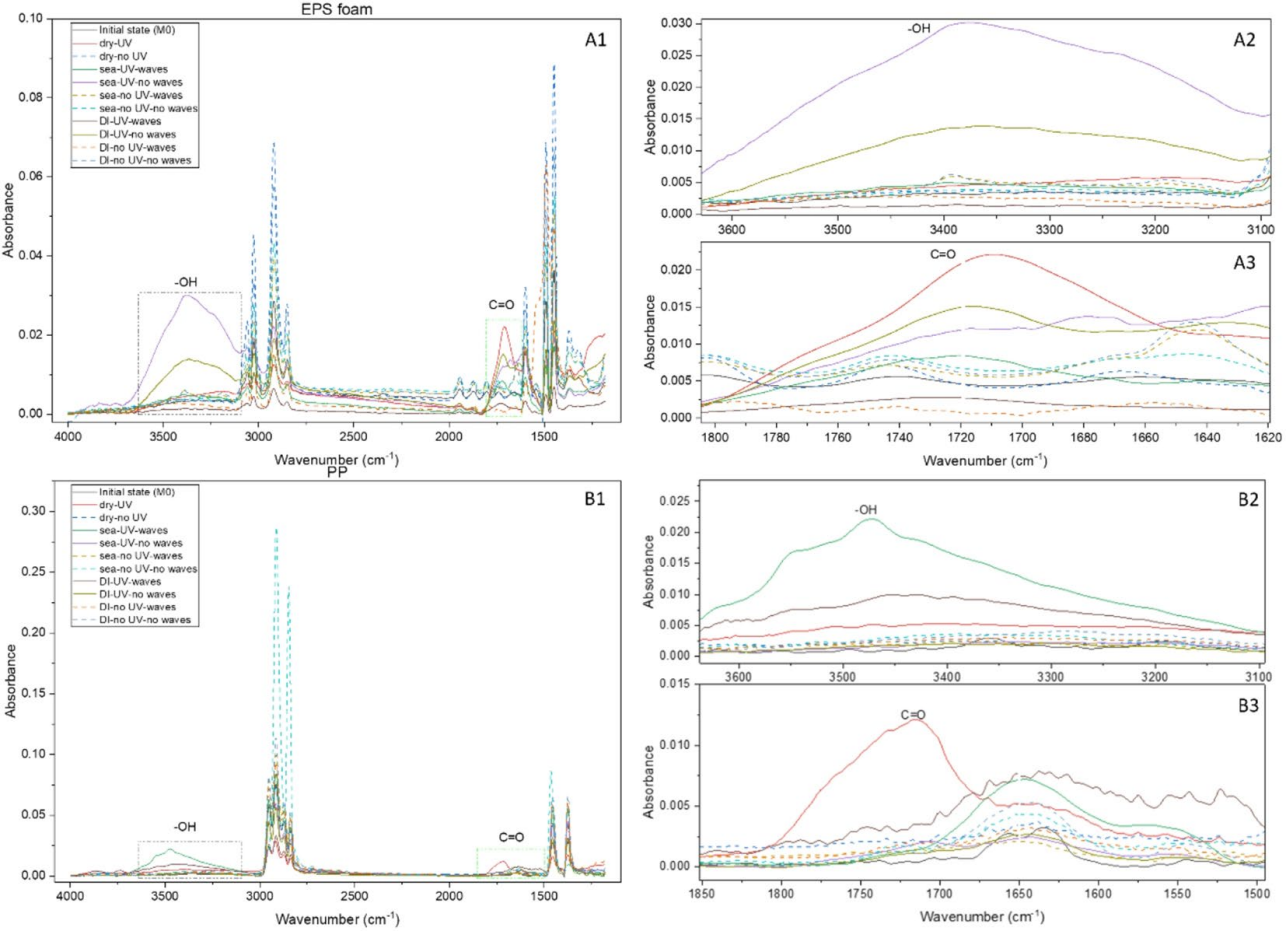
FTIR spectra of EPS foam (A1–A3) and PP bags (B1–B3) after six months of weathering under UV exposure in dry and wet conditions (with and without waves). A1 and B1 show full spectra; A2 and B2, O–H stretching (~ 3300 cm⁻¹); A3 and B3, C = O stretching (~ 1700 cm⁻¹). Individual spectra for each condition are in Supplementary Figs. S1–S2.

The chemical composition of the unweathered EPS foam and PP was confirmed using FTIR and the instrument’s database. Weathered plastic surfaces illustrate the distinction of chemical signatures from the unweathered plastic polymers as shown in Fig. 6. Characteristic absorbance peaks of unweathered EPS foam are apparent at wavenumber 3058, 2979, 1598, 1491 and 1448 cm^− 1^. These peaks correspond to the native bonds of polystyrene polymer. FTIR spectra of unweathered PP plastic bags show peaks at wavenumber 2979, 2873, 1436 and 1374 cm^− 1^, which correspond to the native bond of polypropylene polymer. Furthermore, peaks appearing at wavenumber 3362 and 1633 cm^− 1^ are those of additives used for increasing an outdoor lifetime, which are antioxidant and UV stabilizer, respectively^25,39,40,50^ .

As shown in Fig. 6, the FTIR of both weathered EPS foam containers and PP plastic bags after six months of UV irradiation in all dry and wet conditions (with and without water waves) exhibited the formation of new functional groups, represented by peaks at wavenumber 1650–1850 cm^− 1^ and 3250–3600 cm^− 1^, which corresponded to a carbonyl group (C = O stretching), and a hydroxyl group (O-H stretching), respectively. The zoomed-in regions of interest in Fig. 6 further highlight these chemical changes. For a more detailed view of each environmental condition, including individual FTIR spectra and corresponding magnified regions, please refer to Supplementary Fig. S1 and Fig. S2 for EPS foam and PP, respectively. These findings are consistent with previous studies, which reported the appearance of new absorbance peak corresponding to C = O and O-H stretching in FTIR spectra when PS and PP virgin pellets were exposed to UV irradiation^32,44,51–55^.

Plastics with a carbon-carbon backbone (EPS foam and PP) are susceptible to photo-oxidative degradation. UV radiation has sufficient energy to cleave C-C and C-H bonds in the polymer chain. Consequently, free radicals can be generated and then react to oxygen. This leads to autoxidation in the polymer chain, which finally results in random chain scission or crosslinking^56–61^.

In contrast, new absorbance peaks were absent for EPS foam and PP plastic bags without UV irradiation in all dry and wet conditions (with and without water waves). In other words, this can be due to a slow rate of oxidative degradation of plastics in darkness at room temperature (photo-oxidative)^28,53,62^.

**Fig. 7:**
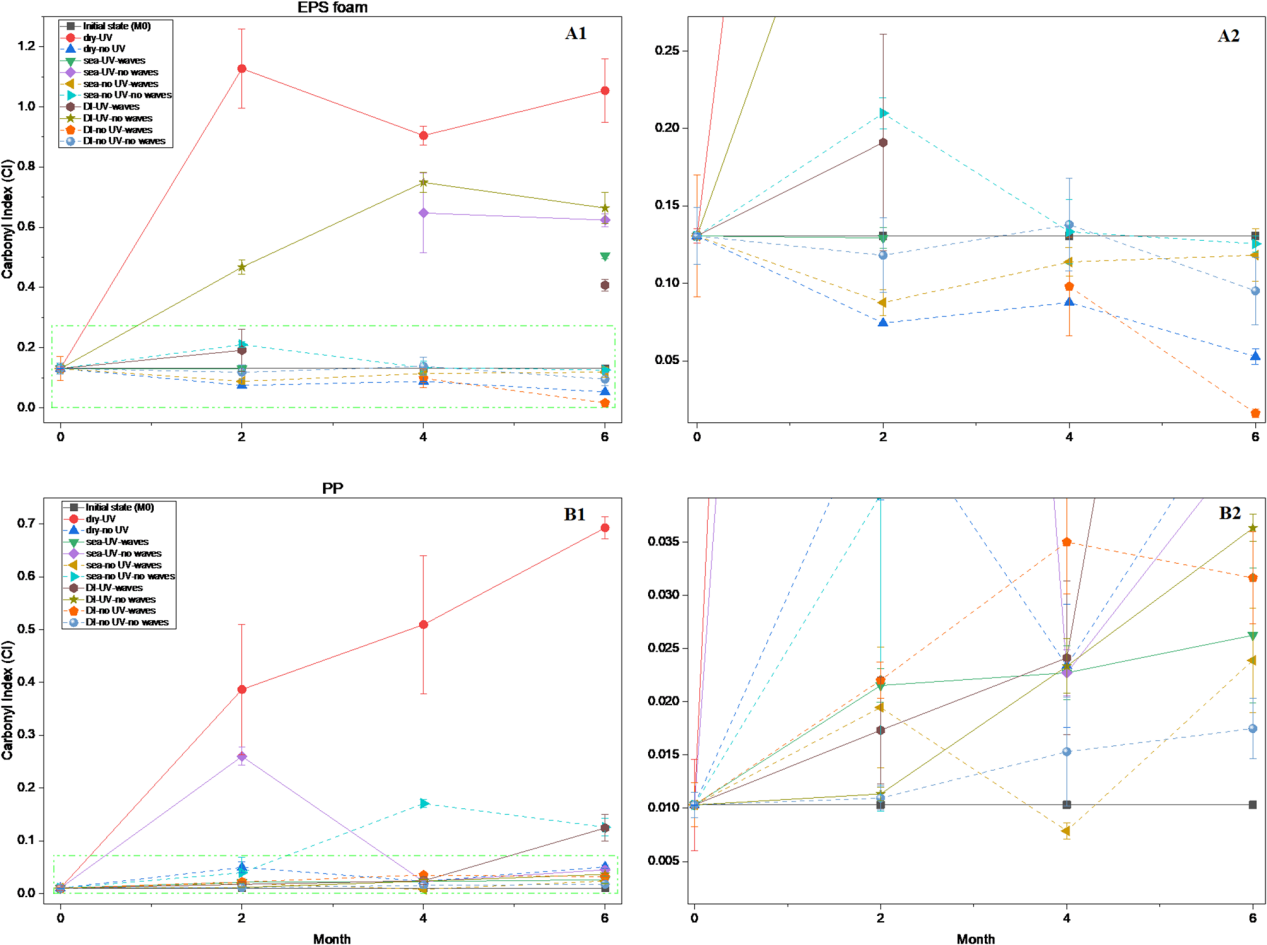
Carbonyl index (CI) of EPS foam and PP during weathering at 0, 2, 4, and 6 months. A1, full EPS trends; A2, EPS zoom-in (low CI range); B1, full PP trends; B2, PP zoom-in (low CI range). Bold solid lines indicate UV exposure; dashed lines indicate non-UV conditions. Values represent mean ± standard deviation (*n* = 3). Four EPS data points are missing due to sample loss/experimental error and are therefore not shown: seawater with UV without water waves (month 2), seawater with UV with water waves (month 4), DI water with UV with water waves (month 4), and DI water without UV with water waves (month 2).

To further quantify the extent of photooxidative degradation, the carbonyl index (CI) was calculated from the FTIR spectra, with results summarized in Fig. 7A1 and A2 for EPS, and Fig. 7B1 and B2 for PP. In this study, EPS foam underwent substantial degradation, with the highest CI of 1.13 observed under dry conditions with UV exposure. In wet environments, peak CI values reached 0.65 and 0.75 under UV exposure but without water waves in seawater and deionized water, respectively. Across all wet conditions, samples exposed without water waves consistently exhibited higher CI values than those with wave agitation.

For PP, the initial CI was low (0.010) and increased to a maximum of 0.69 under dry conditions with UV exposure. In seawater and deionized water, peak CI values were 0.48 (UV without water waves) and 0.12 (UV with water waves), respectively, indicating medium-specific effects of hydrodynamics on oxidation patterns (Fig. 7B1 and B2).

EPS showed a higher CI than PP at month 0, likely from surface oxidation during manufacturing. The steam expansion process for EPS can create oxidation-prone sites on bead surfaces^63^. After six months of UV exposure, EPS maintained higher CI values than PP under nearly all conditions (Fig. 7A panels compared to B panels). The formation of carbonyl groups and unsaturated double bonds contributes to the progressive degradation of plastic polymers. Consequently, the lower CI observed in aged PP suggests a reduced degree of degradation over the long term^41,44,62,64,66^. These comparisons confirm that EPS, particularly in its foam form, is more prone to photooxidation than PP, and that degradation behavior is strongly influenced by polymer composition, physical structure, and environmental exposure conditions.

Compared to previous studies, CI values in this study fall within a comparable range, though they are somewhat lower under similar conditions. Similar to this study, ^39^ investigated expanded polystyrene (EPS) and PP pellets, reporting maximum CI values of ~ 5.8 for EPS foam and ~ 1.0 for PP under UV exposure in dry conditions combined with mechanical abrasion using sand. In contrast, most other studies have examined solid polystyrene (PS), which typically exhibits greater oxidative stability. For instance, ^67^ reported CI values of ~ 2.1 for solid PS and ~ 1.3 for PP under UVA exposure in artificial seawater. ^68^ observed PP CI values up to ~ 1.9 under intensified aging conditions (UVA 12 W/m², 75% RH), while^69^ more recently reported CIs of ~ 0.44–0.56 for micro-PP and only ~ 0.01–0.02 for micro-PS after 180 days of UVA exposure in seawater.

## Discussion

Exposure to UV radiation is a major factor affecting the degradation of polymers^44,70–72^. Inland plastic waste mismanagement plays an important influencing factor of microplastic contamination in urban freshwater and ocean^73–75^. The inland plastic waste enters the river systems through wind and stormwater runoff and ends up in the oceans. The plastic waste exposed to UV radiation in the natural environments (i.e., lithosphere, and hydrosphere) cause photooxidative degradation of polymers that results in the breaking of polymer chains, and the formation of free radicals and other functional groups (i.e., carbonyl group, and hydroxyl group), causing brittle surface, surface cracks and fractures to microplastics^29,32,39,41,70^.

The differences in degradation between EPS foam and PP plastic bags observed in this study are largely attributed to intrinsic material properties. Compared to PP, EPS foam exhibited more advanced degradation under all UV-exposed conditions, primarily due to its lower bond dissociation energy and its distinctive open, porous structure. This mesoporous morphology enables greater UV light penetration and enhances oxygen diffusion throughout the material, both of which accelerate photooxidative reactions and promote surface deterioration. PP, in contrast, possesses a semi-crystalline structure and a denser molecular arrangement, which limits the diffusion of oxygen and UV penetration into the polymer matrix^39,77,78^. Additionally, the higher bond dissociation energies within its saturated hydrocarbon backbone make it more resistant to UV-induced chain scission and oxidation. This explains the milder morphological and chemical changes seen in PP, particularly in wet and dark environments^70,76,77^. It should be noted that the EPS foam containers and PP plastic bags used in this study were commercially produced and may contain additives such as UV stabilizers or antioxidants, which are known to slow down photooxidative degradation by inhibiting chain scission and radical propagation^79,80^. Since the specific additive compositions were not analyzed, some differences in degradation behavior may reflect the presence of such stabilizers. Future studies should include additive profiling to better understand their role in weathering resistance.

Environmental conditions strongly influenced degradation. Dry settings promoted faster photooxidation than wet ones due to greater direct UV exposure with minimal scattering or absorption^64^. In seawater, salts, organic compounds, and suspended particles increase UV scattering and absorption, reducing penetration^81^. EPS foam and PP both float due to density < 1 g/cm³ ^82,83^, sustaining UV exposure at the surface; both are among the most common plastics found in aquatic environments^26,84^. In dark conditions, degradation was minimal, consistent with landfill observations^85^.

Water wave is one of factors affecting the mechanical degradation of polymers under the influence of shear forces that result in the formation of free radicals and other functional groups, causing brittle surface and surface cracks^39,41,44,86^. Even without UV exposure, EPS foam subjected to seawater agitation exhibited early surface cracking and wrinkling, suggesting that mechanical factors alone can initiate degradation. Continuous hydrodynamic shear, turbulence, and abrasion likely imposed repetitive stress and weakening its structure. This supports the role of mechanical forces in initiating or accelerating degradation of structurally fragile plastics like EPS foam in dynamic aquatic environments. In contrast, PP displayed limited mechanical degradation, with small cracks observed only when both UV and seawater waves were applied.

A clear distinction was observed between EPS degradation under dry and wet UV conditions. In dry environments, UV exposure mainly caused surface cracking via photooxidation, whereas wet UV conditions led to more fragmentation. Water likely facilitated breakdown by infiltrating the polymer matrix, causing slight swelling or plasticization that reduced cohesive strength and increased vulnerability to mechanical stress^35,39,87^. Oxidized polymers may also fragment more readily in water due to stress cracking and hydrodynamic shear, even when oxidation is more advanced in dry conditions^43,88,89^. These effects explain the greater fragmentation in wet UV and support the conclusion that PP, with its denser and more chemically resistant structure, requires stronger combined stressors for breakdown.

The rotational agitation used in this study (150 rpm; ~33 m/min) simulated a high-energy aquatic environment but does not fully represent natural marine hydrodynamics. The selected speed, based on three times the maximum average wave velocity in the Gulf of Thailand, was intended to accelerate degradation for analytical observation within the limited timeframe. While constant agitation does not capture intermittent shear, turbulence, or sediment interactions, it enables controlled and reproducible comparisons, providing useful insights into degradation under intensified laboratory conditions.

Another limitation is the absence of mechanical evaluation of embrittlement. Although SEM and FTIR indicated substantial surface deterioration and oxidation in weathered EPS and PP, direct mechanical testing (e.g., tensile strength, elongation at break) was not performed. Future work should incorporate such analyses to quantitatively confirm embrittlement and link it to structural integrity^39,44^.

Furthermore, post-experimental water analysis to detect suspended microplastics or polymer fragments was also not conducted. This study focused on physicochemical degradation of the plastics under controlled conditions, prioritizing surface and structural analyses (SEM, FTIR, CI). Given the small scale and absence of sedimentation or filtration systems, recovery of low concentrations of released particles was not feasible. Similarly, quantitative fragmentation analysis (fragment counting, mass loss, size distribution) was not performed. Future work should include water-phase monitoring and quantitative fragmentation to better assess secondary microplastic release. In addition, variations in degradation across different regions of the same weathered surface may limit consistency in interpretation. Future studies should apply quantitative surface characterization (e.g., image analysis, crack density mapping, profilometry) to strengthen morphological assessments and reproducibility.

Despite the limitations discussed above, the research findings have clear practical relevance. Travel time from major cities to coastal zones is often only days^90^, enabling much mismanaged waste to fragment rapidly upon reaching the sea. Plastics exposed on land before entering waterways are also significant sources of microplastics, consistent with the degradation observed under dry UV conditions. Degradation behavior is governed by polymer structure, morphology, and environmental exposure; understanding these factors is essential for predicting microplastic generation and informing mitigation strategies.

Effective integrated waste management—including collection, sorting, transport, and utilization—is critical to reducing microplastic inputs. While bans and restrictions can help^91^, circular economy approaches offer broader, long-term solutions. By promoting reuse and material recovery, microplastic generation can be minimized. Governments have advanced 3R strategies (Reduce, Reuse, Recycle)^92^, but embedding these in a circular economy—where waste becomes a resource—enhances sustainability. Innovative collection and separation systems improve 3R implementation and resource recovery, reducing environmental leakage^93,94^. Organic waste can be diverted for composting or energy, non-biodegradable materials recycled or upcycled, and combustibles used as alternative fuels. Coupled with circular economy principles, these strategies provide an environmentally and economically viable approach to addressing microplastic pollution^87,95^.

## Conclusion

In this study, weathering experiments were conducted on EPS foam containers and PP plastic bags over six months under ten simulated environmental conditions. EPS foam exhibited significant physical and chemical degradation under UV exposure, including discoloration, surface cracking, and fragmentation, particularly in wet conditions. PP showed less pronounced changes, with fragmentation occurring primarily under dry UV conditions and in seawater with UV exposure and water waves. For both materials, weathering was minimal under dark conditions. SEM and FTIR analyses confirmed material-specific degradation patterns, with EPS foam exhibiting more advanced surface deterioration and higher carbonyl indices than PP under comparable conditions.

These findings underscore the critical role of UV radiation and water dynamics in accelerating the degradation of common plastic products into microplastics. The results highlight the urgent need for robust waste management systems to prevent plastic leakage into the environment, particularly into aquatic systems in coastal and urban areas. Implementing strategies grounded in circular economy principles—such as reducing single-use plastic consumption, enhancing product design for reuse, and improving plastic separation and recycling—can significantly mitigate the formation of secondary microplastics. Additionally, promoting the 3R framework (Reduce, Reuse, Recycle) through public awareness, regulatory support, and infrastructure investment is essential for reducing plastic pollution. By integrating both upstream and downstream interventions, society can more effectively address the root causes of microplastic contamination and minimize its long-term impacts on ecosystems.

## Supplementary Information

Below is the link to the electronic supplementary material.

Supplementary Material 1

Supplementary Material 2

Supplementary Material 3

Supplementary Material 4

## Data Availability

All data generated or analyzed during this study are included in this article and its Supplementary Information file.
