# Supplementary figures and images for: Simulated environmental weathering of expanded polystyrene foam and polypropylene under UV and wave agitation
Source: Sci Rep. 2025 Nov 4;15:38649. doi: 10.1038/s41598-025-22367-7 (PMC12586459; doi:10.1038/s41598-025-22367-7)

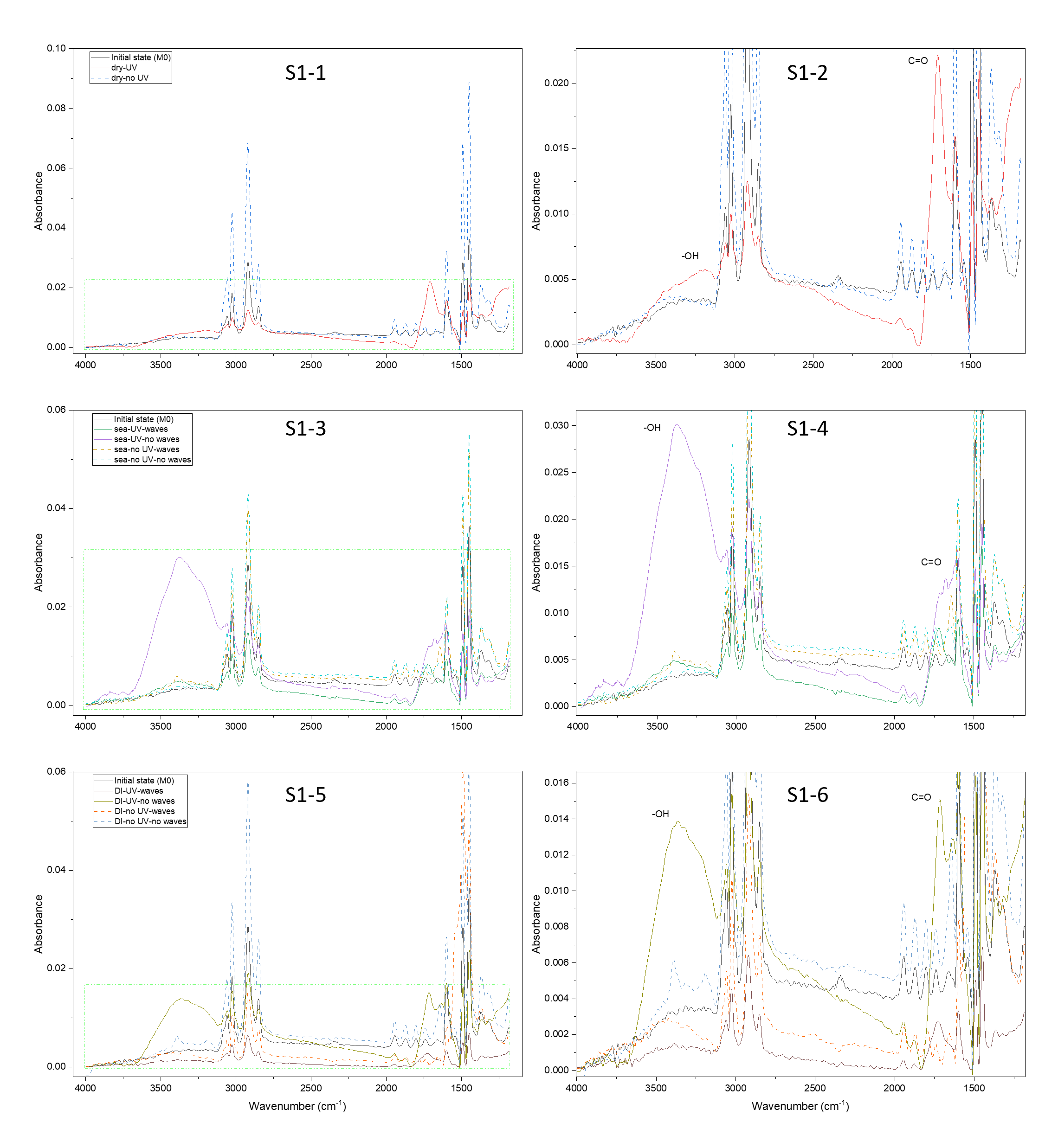

Supplement: Supplementary file 1 — Supplementary Material 1 [file 41598_2025_22367_MOESM1_ESM.tiff]

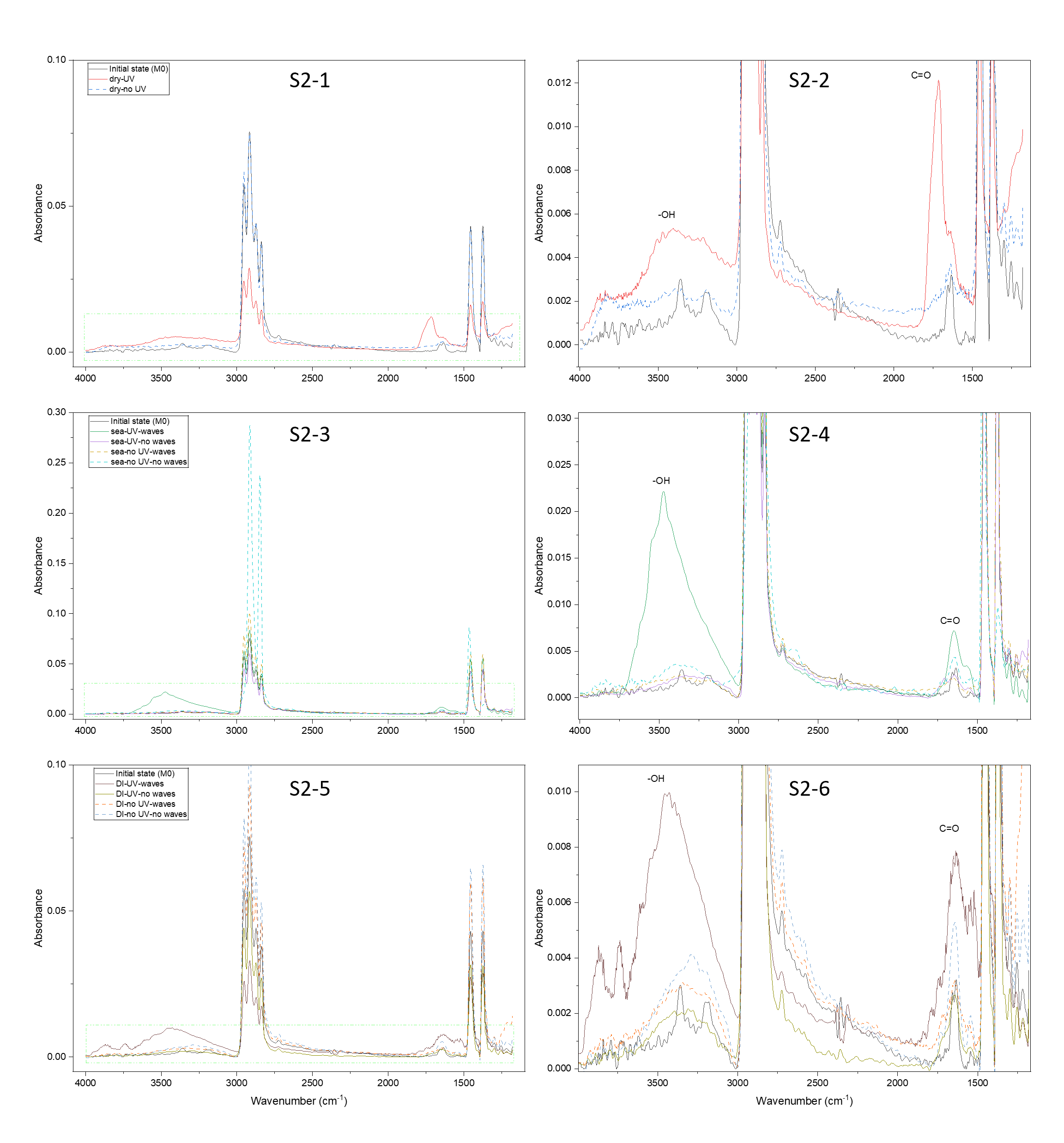

Supplement: Supplementary file 2 — Supplementary Material 2 [file 41598_2025_22367_MOESM2_ESM.tiff]
