# Supplementary material for: Simulated environmental weathering of expanded polystyrene foam and polypropylene under UV and wave agitation
Source: Sci Rep. 2025 Nov 4;15:38649. doi: 10.1038/s41598-025-22367-7 (PMC12586459; doi:10.1038/s41598-025-22367-7)
Supplement: Supplementary file 3 — Supplementary Material 3 [file 41598_2025_22367_MOESM3_ESM.pdf]

Simulated Environmental Weathering of Expanded Polystyrene Foam and Polypropylene under UV and Wave Agitation

Sucheela Polruang, Varinporn Asokbunyarat*, Phichayut Bouthong, Fauzul Rizqa, Anchisa Somprasong

Department of Environmental Engineering, Faculty of Engineering, Kasetsart University, Bangkok 10900, Thailand

* Corresponding author, email: varinporn.a@ku.th

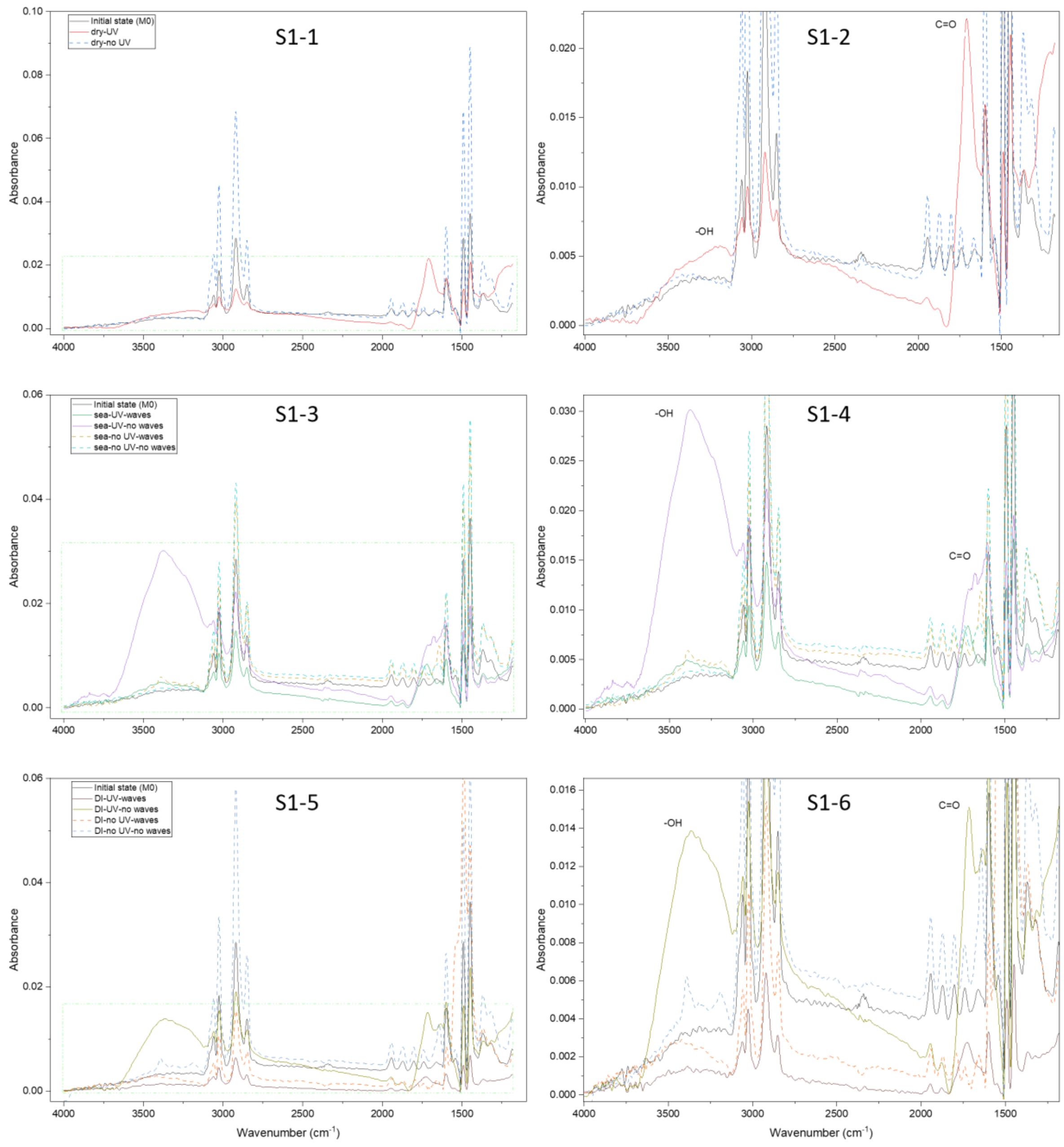

Fig. S1. FTIR spectra of EPS foam after six months of weathering under UV in dry, seawater, and DI water conditions (with and without waves).

S1-1, S1-2: Dry conditions – full spectra; zoom of O–H (~3300 cm^{-1}) and C=O (~1700 cm^{-1}).

S1-3, S1-4: Seawater conditions – full spectra; zoom of O–H and C=O.

S1-5, S1-6: DI water conditions – full spectra; zoom of O–H and C=O.

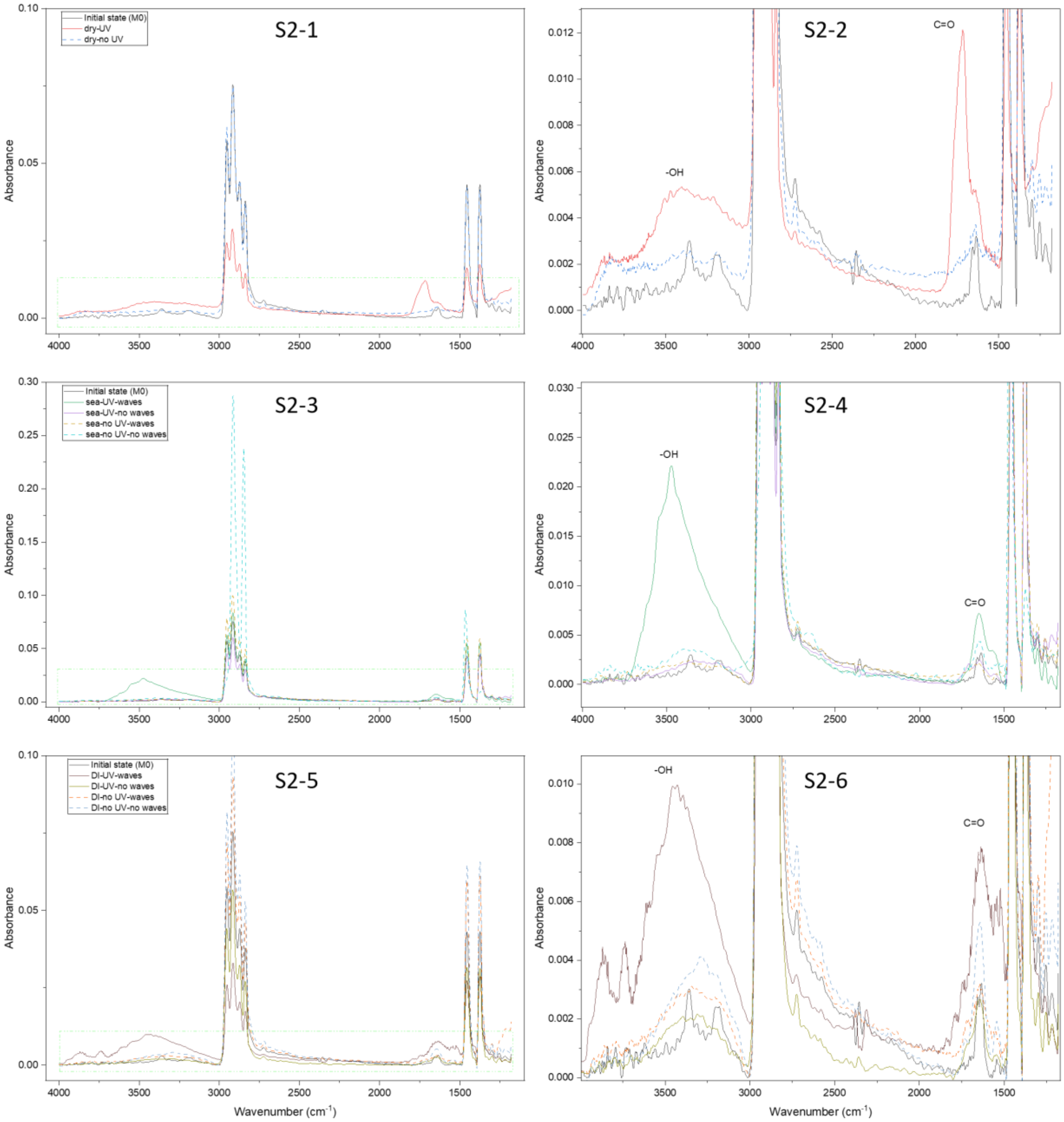

Fig. S2. FTIR spectra of PP after six months of weathering under UV in dry, seawater, and DI water conditions (with and without waves).

S2-1, S2-2: Dry conditions – full spectra; zoom of O–H (~3300 cm^{-1}) and C=O (~1700 cm^{-1}).

S2-3, S2-4: Seawater conditions – full spectra; zoom of O–H and C=O.

S2-5, S2-6: DI water conditions – full spectra; zoom of O–H and C=O.
