# Supplementary material for: Simulated environmental weathering of expanded polystyrene foam and polypropylene under UV and wave agitation
Source: Sci Rep. 2025 Nov 4;15:38649. doi: 10.1038/s41598-025-22367-7 (PMC12586459; doi:10.1038/s41598-025-22367-7)
Supplement: Supplementary file 4 — Supplementary Material 4 [file 41598_2025_22367_MOESM4_ESM.docx]

**Simulated Environmental Weathering of Expanded Polystyrene Foam and Polypropylene under UV and Wave Agitation**

**Sucheela Polruang, Varinporn Asokbunyarat^*^, Phichayut Bouthong, Fauzul Rizqa, Anchisa Somprasong**

*Department of Environmental Engineering, Faculty of Engineering, Kasetsart University, Bangkok 10900, Thailand*

* Corresponding author, email: [varinporn.a@ku.th](mailto:varinporn.a@ku.th)


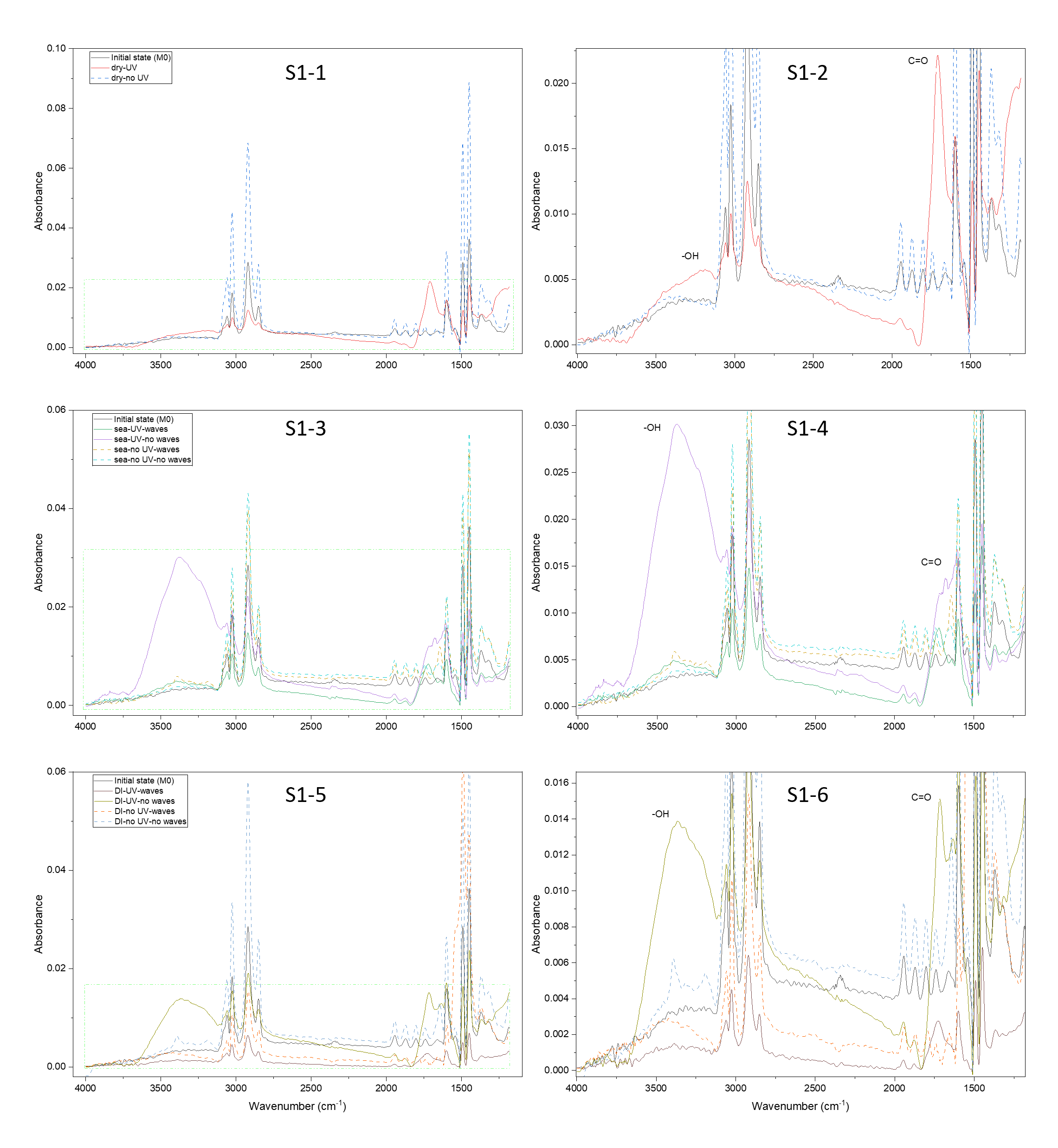


**Fig. S1.** FTIR spectra of EPS foam after six months of weathering under UV in dry, seawater, and DI water conditions (with and without waves).
S1‑1, S1‑2: Dry conditions – full spectra; zoom of O–H (~3300 cm⁻¹) and C=O (~1700 cm⁻¹).
S1‑3, S1‑4: Seawater conditions – full spectra; zoom of O–H and C=O.
S1‑5, S1‑6: DI water conditions – full spectra; zoom of O–H and C=O.


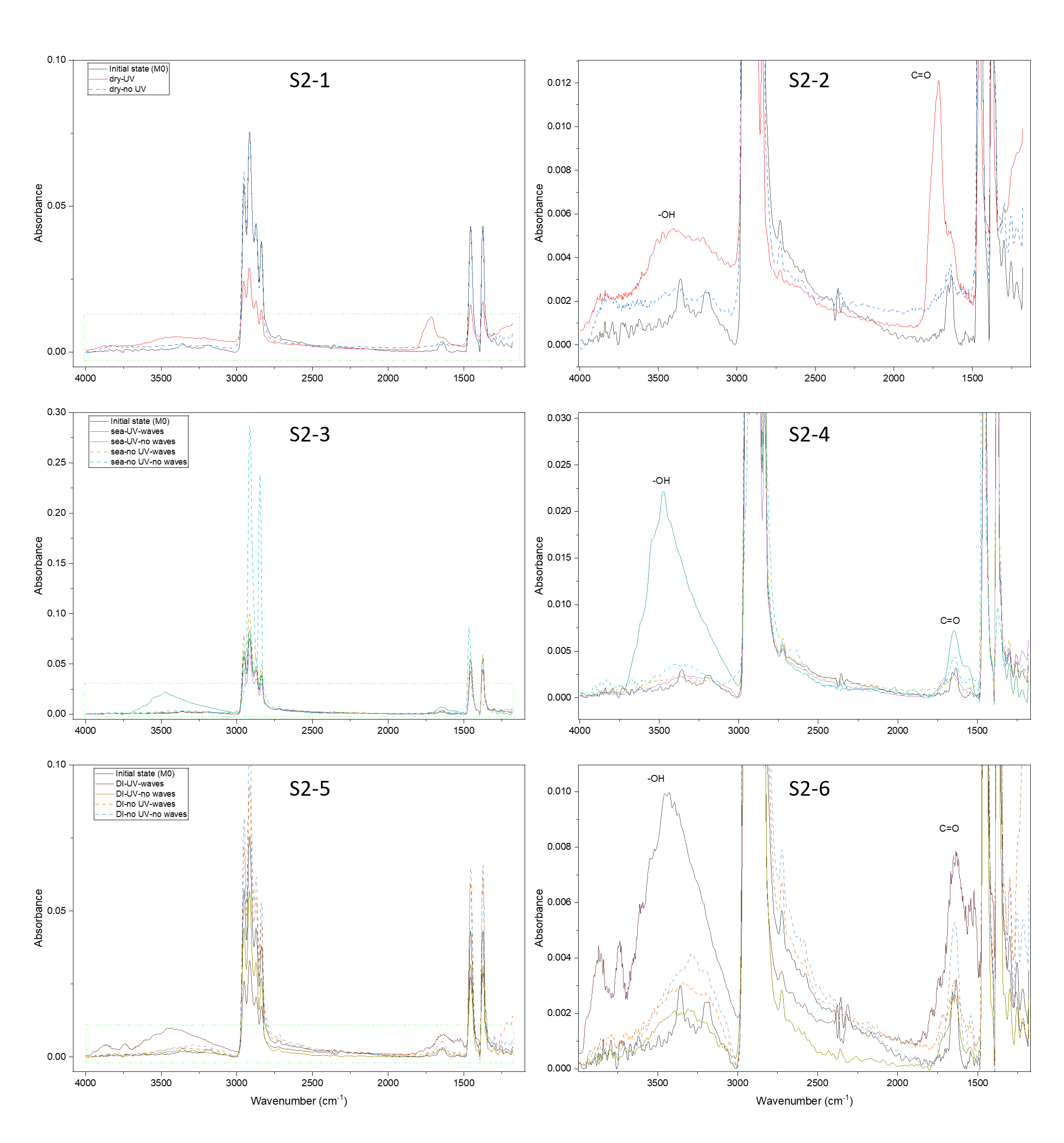


**Fig. S2.** FTIR spectra of PP after six months of weathering under UV in dry, seawater, and DI water conditions (with and without waves).

S2-1, S2-2: Dry conditions – full spectra; zoom of O–H (~3300 cm⁻¹) and C=O (~1700 cm⁻¹).

S2-3, S2-4: Seawater conditions – full spectra; zoom of O–H and C=O.

S2-5, S2-6: DI water conditions – full spectra; zoom of O–H and C=O.
